# The efficacy of taurolidine lock solution in preventing catheter-related bloodstream infections in pediatrics: a systematic review and meta-analysis

**DOI:** 10.3389/fphar.2026.1938862

**Published:** 2026-10-21

**Authors:** Congyu Qin, Bingqiang Huang, Zhiqi Liu, Lingling Huang, Huixiu Liao, Jieru Quan, Liwei Li, Xiaomeng Huang, Weiming Liang

**Affiliations:** 1 The First Affiliated Hospital of Guangxi University of Science and Technology, Guangxi University of Science and Technology, Liuzhou, Guangxi, China; 2 Department of General Surgery, The People’s Hospital of Cenxi City, Cenxi, Guangxi, China; 3 School of Economics and Management, Guangxi University of Science and Technology, Liuzhou, Guangxi, China; 4 Medicine College, Guangxi University of Science and Technology, Liuzhou, Guangxi, China

**Keywords:** catheter-related bloodstream infection, central venous access device, lock, meta-analysis, pediatrics, taurolidine

## Abstract

**Background:**

This systematic review and meta-analysis evaluated the efficacy and safety of taurolidine lock solutions in preventing catheter-related bloodstream infections in pediatric patients with central venous access devices.

**Methods:**

This meta-analysis systematically searched four electronic databases to identify studies comparing taurolidine with standard lock solutions for catheter lock in pediatric patients. The primary outcome measure was the incidence density of catheter-related bloodstream infections. Secondary outcomes included the incidence density of central line-associated bloodstream infection, cumulative incidence of catheter-related bloodstream infections, catheter removal due to infection, central venous access device (CVAD)-related thrombosis, and adverse events. Random-effects models were used to pool incidence rate ratios (IRRs) for incidence-density outcomes and risk ratios (RRs) for dichotomous outcomes, with corresponding 95% confidence intervals.

**Results:**

In the primary analysis of five studies, taurolidine-containing catheter lock solutions were associated with a significantly lower incidence rate of catheter-related bloodstream infection (CRBSI) compared with control solutions (IRR = 0.23, 95% CI: 0.15–0.36; I^2^ = 0%; P < 0.00001). The separate secondary analysis of three studies also showed a lower incidence density of central line-associated bloodstream infection (CLABSI), although substantial heterogeneity was observed (IRR = 0.25, 95% CI: 0.08–0.85; I^2^ = 90%; P = 0.03). The cumulative incidence of catheter-related bloodstream infections was lower in the taurolidine group (RR = 0.37, 95% CI: 0.23–0.61; I^2^ = 54%; P = 0.0001). No statistically significant differences were detected in catheter removal due to infection (RR = 1.25, 95% CI: 0.60–2.62; I^2^ = 12%; P = 0.56), CVAD-related thrombosis (RR = 0.88, 95% CI: 0.29–2.64; I^2^ = 0%; P = 0.82), or adverse events (RR = 3.93, 95% CI: 0.82–18.80; I^2^ = 64%; P = 0.09).

**Conclusion:**

Taurolidine-containing lock solutions may reduce the incidence density and cumulative incidence of CRBSI in pediatric patients requiring central venous access. However, the available evidence is insufficient to establish their comparative safety. Given the substantial heterogeneity, mixed study designs, variability in infection definitions, and limited safety data, these findings should be interpreted cautiously. Further large-scale, multicenter randomized controlled trials with adequate safety monitoring are needed before broad routine implementation can be recommended.

**Systematic Review Registration:**

https://www.crd.york.ac.uk/prospero/display_record.php?ID=CRD420251162414 identifier, CRD420251162414.

## 1 Introduction

The central venous access device (CVAD) is fundamental to contemporary pediatric critical care and the management of complex diseases. Its clinical value and the prevention of associated complications have always been the focus of medical attention. In the context of cancer chemotherapy, management of primary immunodeficiency, parenteral nutrition infusion and intensive care (PICU/NICU), CVADs effectively address treatment challenges posed by specific vascular conditions, such as vascular fragility and critical circulatory instability in children with low body weight, by establishing stable central venous access (Curtis et al., 2025). The infusion of cytotoxic drugs via a central venous access device, frequent blood sample collection, or hypertonic nutrition infusion can markedly diminish tissue damage and suffering associated with repeated peripheral punctures (Johansson et al., 2013; Bahoush et al., 2021). Nonetheless, the creation of this “lifeline” is fraught with the potential for significant complications, with catheter-related bloodstream infection (CRBSI) emerging as a major clinical complication in pediatric central venous access device management, attributable to its elevated incidence, intricate treatment, and lethality (Saliba et al., 2018).

Catheter-related bloodstream infection, a significant consequence of central venous access, frequently occurs in individuals with severe illnesses or hematological malignancies and those receiving parenteral nutrition (Wouters et al., 2019). Microorganisms can colonize catheters through several routes. Skin organisms may migrate from the insertion site along the external catheter surface (extraluminal route), whereas contamination of catheter hubs or needleless connectors may introduce microorganisms into the lumen and promote intraluminal biofilm formation. Less commonly, hematogenous spread from a distant infectious focus may lead to secondary catheter colonization (Di Domenico et al., 2022). The prevalent organisms responsible for catheter-related bloodstream infections include coagulase-negative staphylococci, Enterobacteriaceae, *Pseudomonas aeruginosa*, and *Candida* (Saliba et al., 2018; Tekeli et al., 2025). The adverse consequences of CRBSI include persistent bacteremia and sepsis, leading to disseminated infections such as endocarditis, osteoarticular infections, and pneumonia. It is also linked to catheter thrombosis, catheter failure, prolonged hospital stays, escalating medical expenses, and an elevated mortality risk (Chong et al., 2013).

In recent years, taurolidine lock solution has been widely used in local interventions for closing the catheter lumen. The taurolidine lock solution chemically interacts with bacterial cell wall components, including endotoxins and lipopolysaccharides, compromising the bacterial membrane structure and thereby demonstrating broad-spectrum antibacterial, antifungal, and anti-endotoxin properties (Caruso et al., 2010; Olthof et al., 2015). Taurolidine, unlike conventional antibiotics, does not achieve its bactericidal effect through the inhibition of protein synthesis or DNA replication, hence reducing the likelihood of resistance development and conferring distinct advantages in anti-infection applications (Liu et al., 2013). Nonetheless, there exists significant variability in the evidence concerning the efficacy and safety of taurolidine lock in pediatrics: certain randomized controlled trials indicate that clinical bacterial isolates in the taurolidine lock cohort exhibit no resistance, and even high-dose intravenous administration demonstrates no apparent toxicity (Simon et al., 2008). However, concerns remain regarding pathogen-specific effects, fungal infections, and potential microbial selection during long-term use, although current evidence has not consistently demonstrated an increased risk. Previous systematic reviews and meta-analyses have also emphasized that the certainty of evidence is limited by small sample sizes, heterogeneous populations, variable lock formulations, and inconsistent infection definitions. These uncertainties underscore the need for an updated and rigorous reassessment of the available pediatric evidence (Liu et al., 2013; Van Den Bosch et al., 2024). Sun et al. (2020) suggested that taurolidine can be used as a lock solution for preventing central venous catheter-related bloodstream infections in pediatric patients, but the number of included studies is limited and the strength of the evidence is insufficient. Given the recent proliferation of studies, an updated systematic review and meta-analysis of taurolidine utilization in pediatric populations is essential to derive more comprehensive and valid results.

## 2 Materials and methods

### 2.1 Search strategy

This meta-analysis was conducted according to the preferred reporting items for systematic reviews and meta-analyses (PRISMA) statement (Moher et al., 2009) and the Cochrane Handbook for interventional systematic reviews (Higgins and Green, 2012). This meta-analysis has been registered on Prospero website with the registration number PROSPERO (CRD420251162414). This meta-analysis systematically searched PubMed, Embase, Web of Science, and the Cochrane Library for eligible studies published up to 13 September 2026. The search strategies combined controlled vocabulary, including Medical Subject Headings (MeSH) where applicable, with free-text terms related to taurolidine lock solutions, central venous access devices, catheter-related bloodstream infections, and pediatric populations.

### 2.2 PICOS standard construction

This meta-analysis used the PICOS (study population, interventions, controls, outcome indicators and study design) principle to be included in the study. The inclusion criteria for each dimension are as follows:

1. Research population: pediatric patients who need long-term intravenous catheters for any reason.
2. Interventions: any taurolidine-containing catheter lock.
3. Control measures: conventional catheter lock (such as heparin saline).
4. Outcome measures:
  1. Main outcome measures: Incidence density of catheter-related bloodstream infections;
  2. Secondary outcome measures: Incidence density of central line-associated bloodstream infection; cumulative incidence of catheter-related bloodstream infection, number of catheter removal due to infection, CVAD-related thrombosis and adverse events.
5. Study design type: randomized controlled trial (RCT) or non-randomized controlled trial.
6. Exclusion criteria include:
  1. Other types of articles, such as case reports, abstracts, publications, letters, reviews, meta-analyses, editorials, animal studies and protocols.
  2. Not relevant.
  3. Other drugs, such as antibiotic or minocycline–ethylenediaminetetraacetic acid (M-EDTA).
  4. Fail to obtain full study report due to inaccessibility of the article.
  5. Reduplicate cohort of patients.
  6. Fail to extract data.

There was no language restriction.

### 2.3 Other literature sources

This meta-analysis examined the references of the included articles and incorporated the literature that satisfied the inclusion criteria of this study in the systematic review and meta-analysis. Any disagreements regarding the eligibility of additionally identified studies were resolved through discussion.

### 2.4 Data extraction

After duplicate removal, two reviewers (ZL and LH) independently screened the titles and abstracts of the retrieved records. The full texts of potentially eligible articles were then independently assessed by the same reviewers against the predefined eligibility criteria. Reasons for exclusion at the full-text stage were recorded. Disagreements during study selection or data extraction were resolved through discussion and, when consensus could not be reached, consultation with a third reviewer (CQ). Data from the included studies were independently extracted by the same two reviewers using a prespecified form. The extracted information included the first author, publication year, study period, study design, country, participant characteristics, sample size, duration of catheter use, lock protocol, study outcomes, and complications. The extraction form was developed before data extraction but was not formally piloted. The review protocol specified that the corresponding authors would be contacted by email when relevant data were missing or could not be extracted.

### 2.5 Outcome definitions

The primary efficacy outcome was the incidence density of catheter-related bloodstream infection, expressed as the number of CRBSI episodes per 1,000 catheter-days. For the primary analysis, CRBSI was restricted to laboratory-confirmed bloodstream infections for which microbiological findings supported the CVAD as the source, based on the diagnostic criteria applied in each original study.

Central line-associated bloodstream infection (CLABSI) was treated as a distinct secondary outcome. CLABSI was defined as a laboratory-confirmed bloodstream infection meeting the central-line association and timing criteria specified by the surveillance definition used in each original study, without attribution to another infection site and without requiring microbiological confirmation that the catheter was the source.

Because CRBSI and CLABSI differ in their diagnostic purposes, degrees of causal attribution, and susceptibility to outcome misclassification, the two definitions were not considered interchangeable and were not combined in the same meta-analysis. Studies reporting CRBSI contributed to the primary analysis, whereas studies explicitly reporting CLABSI contributed to a separate secondary analysis.

For each study, classification was based primarily on the operational diagnostic criteria reported by the original investigators rather than solely on the terminology used in the article. Two reviewers independently assessed the reported infection definitions. Disagreements were resolved through discussion and, when necessary, consultation with a third reviewer. The original terminology and diagnostic criteria were retained in the study-characteristics table to ensure transparency.

### 2.6 Risk of bias assessment

The risk of bias was evaluated by two independent reviewers (ZL and LH), with inconsistencies addressed by communication with a third reviewer (CQ). This meta-analysis employed the Cochrane Collaboration risk assessment methodology (ROB 2) to examine these RCTs (Sterne et al., 2019), encompassing five dimensions (Curtis et al., 2025): Randomization process (Johansson et al., 2013); Deviations from intended interventions (Bahoush et al., 2021); Missing outcome data (Saliba et al., 2018); Measurement of the outcome (Wouters et al., 2019); Selection of the reported result. In this scoring criteria, green signifies low risk, red denotes high risk of bias, and yellow represents some concerns.

For non-RCT research, we employed the Newcastle-Ottawa Scale for bias evaluation (Stang, 2010). The scale encompasses three domains (Curtis et al., 2025): cohort selection (4 items), which assesses the representativeness of the exposure group (1 point), the selection of the non-exposure group (1 point), ascertainment of exposure (1 point), and the outcome of interest was not present at the start of the study (1 point) (Johansson et al., 2013); comparability (2 items), evaluating the comparability of the principal factors (1 point) and the comparability of additional risk factors (1 point) (Bahoush et al., 2021); outcome (3 items), which includes outcome assessment (1 point), adequacy of follow-up duration (1 point), and completeness of follow-up (1 point). Individual item ratings are presented in Table 1. Interpretation focused on the methodological strengths and limitations identified within each domain. No numerical threshold was used to classify studies as high quality, and total NOS scores were not interpreted as equivalent to an overall judgment of low risk of bias.

**TABLE 1:** Quality assessment according to the Newcastle-Ottawa scale.

Author, year	Selection				Comparability		Outcome			Total scores
	Representativeness	Selection of non-exposure	Ascertainment of exposure	Outcome not present at start	Comparability on most important factors	Comparability on other risk factors	Assessment of outcome	Adequate follow-up time	Complete follow-up	
Simon et al. (2008)	*	*	-	*	*	*	*	*	*	8
Ince et al. (2014)	*	-	*	*	*	*	-	*	*	7
Hulshof et al. (2017)	*	*	-	*	*	*	*	*	*	8
Lambe et al. (2018)	*	*	-	*	*	-	*	*	*	7
Clark et al. (2019)	*	-	*	*	*	*	*	*	*	8
Chong et al. (2020)	*	*	-	*	-	*	*	*	*	7

“*”indicates criterion met; “**-**”indicates significant of criterion not.

### 2.7 Statistical analysis

All meta-analyses were performed using Review Manager software (Revman, version 5.3; Nordic Cochrane Centre [Cochrane collaboration], Copenhagen, Denmark; 2014). CRBSI incidence density was designated as the primary efficacy outcome, whereas CLABSI incidence density was analyzed separately as a secondary outcome. CRBSI and CLABSI were not combined in the same incidence-density meta-analysis because their diagnostic definitions and degree of catheter attribution differ. The intervention was defined as any taurolidine-containing catheter lock. The different formulations were pooled because they all contained taurolidine as the intended antimicrobial component and were used for the prevention of catheter-associated bloodstream infections. Accordingly, the pooled analyses were intended to estimate the overall association of taurolidine-containing lock regimens as a class rather than the effect of a single standardized formulation. Differences in taurolidine concentration and additional components, including citrate and heparin, were considered potential sources of clinical heterogeneity. Formulation-specific subgroup analyses were not performed because too few studies contributed to each formulation category to provide reliable estimates.

For incidence-density outcomes, the numbers of distinct bloodstream infection episodes and accumulated catheter-days were extracted separately for the taurolidine and control groups. The incidence rate ratio was calculated as IRR = (E_t_/T_t_)/(Ec/Tc), where E and T represent the number of infection episodes and accumulated catheter-days, respectively, in the taurolidine and control groups. Distinct recurrent episodes occurring in the same patient were counted separately when they were reported as separate infection episodes by the original investigators (Higgins et al., 2024).

The study-specific log-transformed IRRs were pooled using the generic inverse-variance method. When raw infection-event counts and catheter-day data were available, the standard error was calculated under a Poisson assumption as SE [ln (IRR)] = √(1/E_t_ + 1/Ec) (Higgins et al., 2024). When an original study directly reported an IRR and its 95% confidence interval, the reported estimate was used and its standard error was calculated as [ln (upper 95% confidence limit) − ln (lower 95% confidence limit)]/3.92. Studies without sufficient arm-specific catheter-day data or a directly reported IRR were excluded from the incidence-density meta-analysis. None of the studies included in the CRBSI or CLABSI incidence-density analyses had zero infection events in either treatment arm; therefore, no continuity correction was applied to the IRR analyses.

For dichotomous outcomes, including the cumulative incidence of catheter-related bloodstream infection, catheter removal due to infection, CVAD-related thrombosis, and adverse events, study-specific risk ratios (RRs) and corresponding 95% confidence intervals were calculated from the numbers of participants or catheters with an event and the corresponding group totals. The RRs were pooled using the Mantel–Haenszel method. For studies with zero events in one treatment arm, a continuity correction of 0.5 was applied by RevMan. Single-arm zero events occurred in two studies contributing to the adverse-event analysis. Studies with zero events in both treatment arms were considered not estimable and did not contribute to the corresponding risk-ratio analysis.

Random-effects models were used for all meta-analyses because clinical and methodological heterogeneity was anticipated across studies, irrespective of the observed I^2^ value. Between-study variance was estimated using the DerSimonian–Laird method. Statistical heterogeneity was assessed using Cochran’s Q test and quantified using the I^2^ statistic. Leave-one-out sensitivity analyses were conducted separately for the primary CRBSI analysis and the secondary CLABSI analysis by sequentially excluding each study. In addition, a sensitivity analysis restricted to randomized controlled trials was conducted for the primary CRBSI incidence-density outcome. All statistical tests were two-sided, and P < 0.05 was considered statistically significant.

## 3 Results

### 3.1 Included studies

This meta-analysis identified 3,574 records from four databases and additional eligible studies through reference-list screening. After 832 duplicates were removed, 2,742 database records underwent title and abstract screening, of which 2,727 were excluded. A total of 15 studies were assessed, and ultimately 9 studies satisfied the inclusion criteria for the systematic review and were included in the meta-analysis. Figure 1 illustrates the procedure for screening and selecting studies. Supplementary Material S2 lists the articles excluded after full-text assessment and the corresponding reasons for exclusion.

**FIGURE 1:**
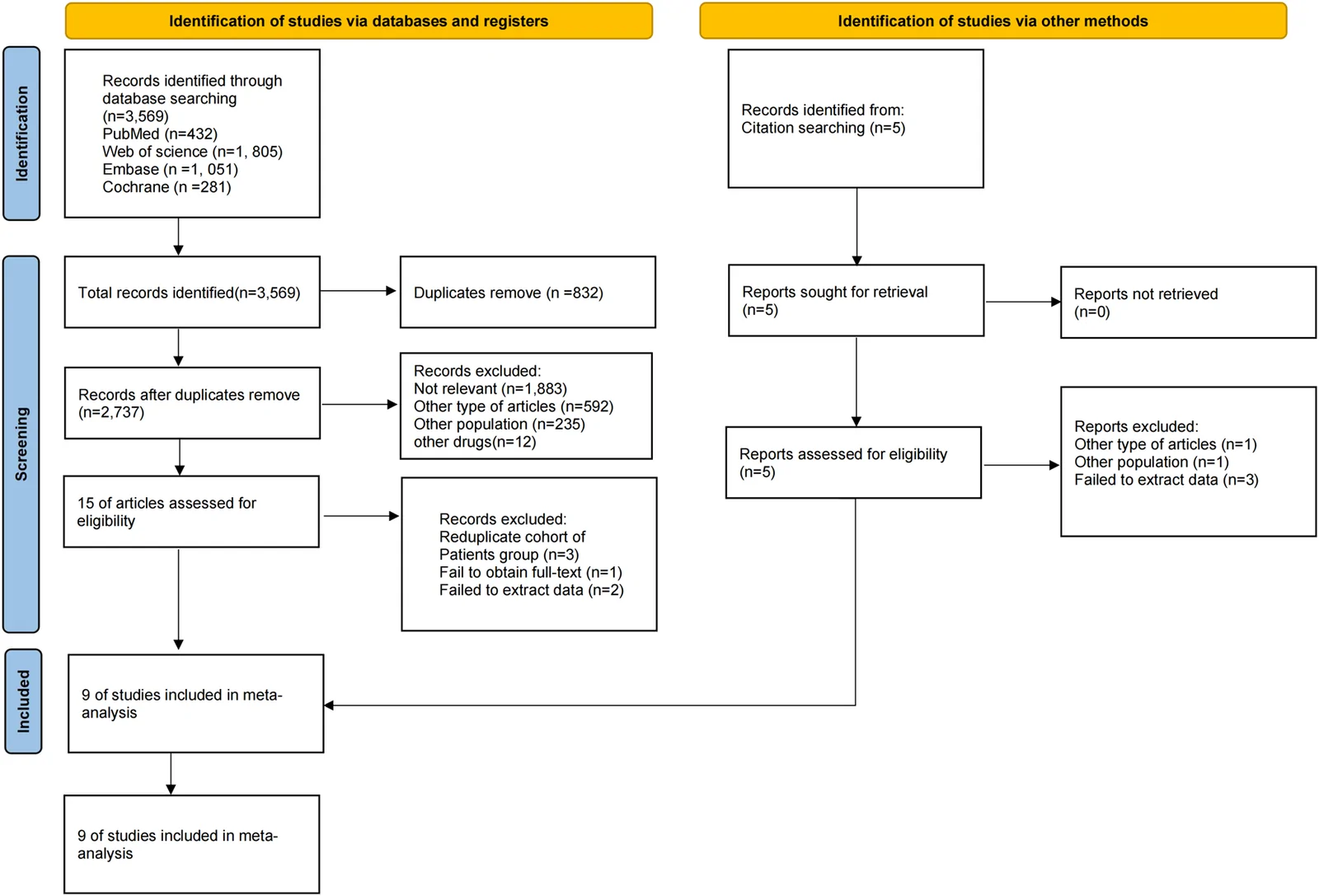
Flow chart of literature search strategies.

### 3.2 Study characteristics

The nine studies included in this meta-analysis were published between 2008 and 2024 and included a total of 1,200 children. The studies were conducted in Germany (2, 22.2%), Denmark (1, 11.1%), the Netherlands (2, 22.2%), France (1, 11.1%), Singapore (1, 11.1%), Turkey (1, 11.1%), and Australia (1, 11.1%). Three were RCTs (Van Den Bosch et al., 2024; Dümichen et al., 2012; Handrup et al., 2013), and six were retrospective studies (Simon et al., 2008; Ince et al., 2014; Hulshof et al., 2017; Lambe et al., 2018; Clark et al., 2019; Chong et al., 2020). Five studies included children with solid tumors or hematological malignancies, whereas the remaining four included children requiring CVADs during or after surgery, for parenteral nutrition or medication administration, or during critical illness. The duration of catheter use varied widely among the included studies. Regarding the lock formulations, five studies used a solution containing 1.35% taurolidine and 4% sodium citrate; one used a solution containing 1.34% taurolidine and 4% sodium citrate; two used a solution containing 1.35% taurolidine, 4% sodium citrate, and 100 IU/mL heparin; and one used 2% taurolidine alone. Eight studies used heparin as the control lock solution, whereas one used saline. Two studies used prophylactic antibiotics during the study. Clinical details of the included pediatric participants are presented in Table 2, whereas details of the interventions and lock protocols are provided in Supplementary Material S3. The risk-of-bias assessments are presented in Figure 2 and Table 1.

**TABLE 2:** Basic characteristics of included trials.

Author, year	Country	Study period	Study design	Patient population	Groups	Sample of patients	Gender (M/F)	Age in years (median) range
Simon et al. (2008)	Germany	2003-2007	Retrospective cohort study	Cancer	Taurolidine	89	60/29	7.2(3.7–16.1)Δ
					Control	90	51/39	10.4(5.2–14.7)Δ
Dümichen et al. (2012)	Germany	2007-2008	RCT	Hematological malignancies	Taurolidine	35	19/16	7.5(1.4–18)
					Control	36	23/13	6.3(1.7–17.1)
Handrup et al. (2013)	Denmark	2008-2010	RCT	Cancer	Taurolidine	64	45/19	6(0–19)
					Control	65	33/32	5(0–16)
Ince et al. (2014)	Turkey	2005-2013	Retrospective cohort study	TIAP	Taurolidine	79	NA	NA
					Control	33	NA	NA
Hulshof et al. (2017)	Netherlands	2008-2012	Retrospective cohort study	HPN	Taurolidine	8	NA	0.23(0–5.25)
					Control	8	NA	0.23(0–5.25)
Lambe et al. (2018)	France	2008-2014	Retrospective cohort study	HPN	Taurolidine	40	24/16	2.2 (1.3–9.6)Δ
					Control	86	41/45	5.7 (1.3–11.5)Δ
Clark et al. (2019)	Australia	2013-2015	Retrospective cohort study	Critically ill children	Taurolidine	19	9/10	6.2(0.3–17)
					Control	19	9/10	6.2(0.3–17)
Chong et al. (2020)	Singapore	2013-2017	Retrospective cohort study	H/O and GI	Taurolidine	33	21/12	3.5(1.5–10.2)Δ
					Control	33	21/12	3.5(1.5–10.2)Δ
Van Den Bosch et al. (2024)	Netherlands	2020-2023	RCT	Oncology	Taurolidine	231	133/98	8(0–18)
					Control	232	134/98	8(0–18)

Δ, IQR, interquartile range; NA, not available; M/F, male/female; TIAP, totally, implantable access port; HPN, home parenteral nutrition; H/O and GI, Hematology–oncology and gastrointestinal; RCT, randomized controlled trial.

**FIGURE 2:**
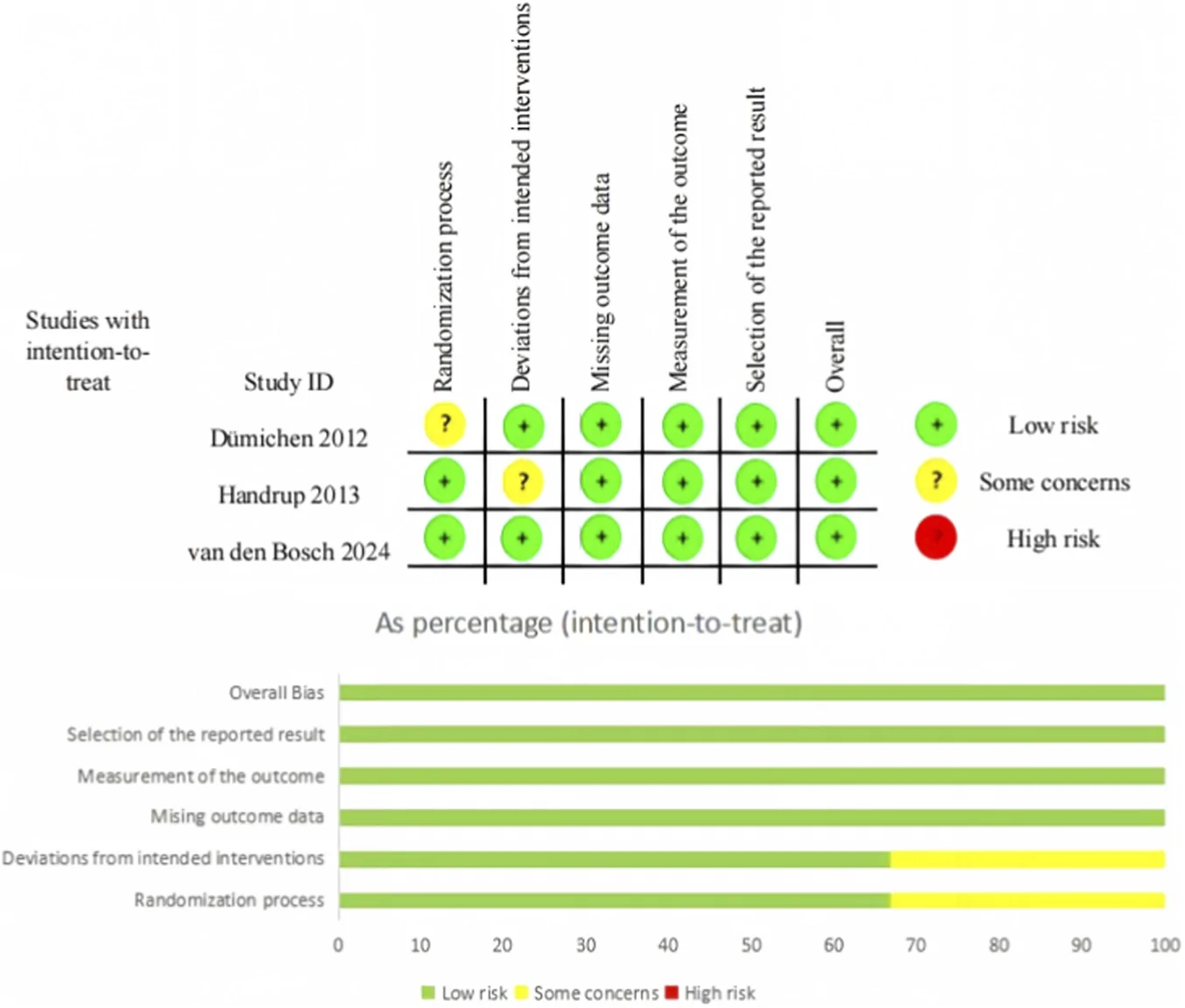
Cochrane Collaboration’s risk of bias assessment diagram.

### 3.3 Outcomes

Table 3 summarizes the results of meta-analyses for all clinical outcomes.

**TABLE 3:** All clinical outcomes.

Outcomes	No. of studies	Study design		Sample size		Heterogeneity		Overall effect size	95% CI of overall effect	P value
		RCT	Retrospective cohort study	Taurolidine	Control	I^2^ (%)	P value			
Incidence density of catheter-related bloodstream infections	5	2	3	236	285	0	0.99	IRR = 0.23	0.15-0.36	<0.00001
Incidence density of central line-associated bloodstream infection	3	1	2	283	284	90	<0.0001	IRR = 0.25	0.08-0.85	0.03
Cumulative incidence of catheter-related bloodstream infection	6	3	3	538	542	54	0.06	RR = 0.37	0.23-0.61	0.0001
Catheter removal due to infection	4	3	1	419	423	12	0.33	RR = 1.25	0.60-2.62	0.56
CVAD-related thrombosis	2	2	0	266	268	0	0.41	RR = 0.88	0.29-2.64	0.82
Adverse events	4	3	1	419	423	64	0.04	RR = 3.93	0.82-18.80	0.09

CI, confidence interval; IRR, incidence rate ratio; RR, risk ratio; CVAD, central venous access device.

#### 3.3.1 Incidence density of catheter-related bloodstream infections

Of the 9 included articles, 5 articles reported the incidence density of catheter-related bloodstream infections (Simon et al., 2008; Dümichen et al., 2012; Handrup et al., 2013; Hulshof et al., 2017; Lambe et al., 2018). Taurolidine-containing lock solutions were associated with a significantly lower incidence density of CRBSI than control lock solutions (IRR = 0.23, 95% CI: 0.15–0.36, I^2^ = 0%, P < 0.00001) (Figure 3). Sensitivity analysis showed stable results (Supplementary Figure S1). The funnel plot appeared broadly symmetrical on visual inspection (Supplementary Figure S2). In the RCT-only sensitivity analysis, the pooled IRR was 0.26 (95% CI 0.11–0.58; I^2^ = 0%; P = 0.001) (Dümichen et al., 2012; Handrup et al., 2013). The individual IRRs were 0.24 (95% CI 0.05–1.13) for Dümichen et al. and 0.26 (95% CI 0.10–0.68) for Handrup et al. (2013) (Supplementary Figure S3).

**FIGURE 3:**
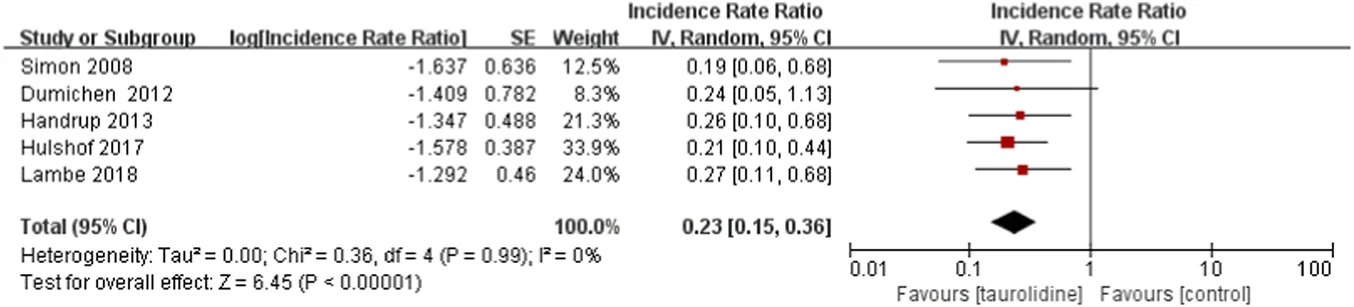
Forest plot of the meta-analysis for incidence density of catheter-related bloodstream infections.

#### 3.3.2 Incidence density of central line-associated bloodstream infection

Of the 9 included articles, 3 articles reported the incidence density of central line-associated bloodstream infection (Van Den Bosch et al., 2024; Clark et al., 2019; Chong et al., 2020). The results showed that the incidence density of central line-associated bloodstream infection was significantly lower than the control group (IRR = 0.25, 95% CI: 0.08–0.85, I^2^ = 90%, P = 0.03) (Figure 4). Substantial between-study heterogeneity was observed. The leave-one-out sensitivity analysis indicated that the statistical significance of the pooled estimate was sensitive to the omission of individual studies (Supplementary Figure S4). The funnel plot for CLABSI appeared visually asymmetric (Supplementary Figure S5).

**FIGURE 4:**
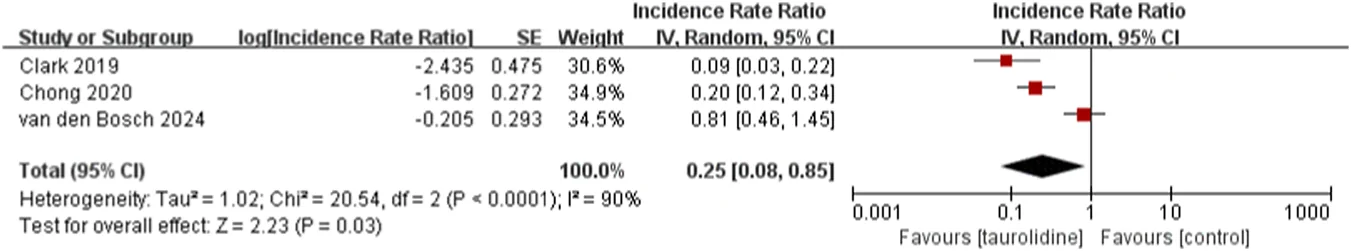
Forest plot of the meta-analysis for incidence density of central line-associated bloodstream infection.

#### 3.3.3 Cumulative incidence of catheter-related bloodstream infection

Of the 9 included studies, 6 reported the cumulative incidence of catheter-related bloodstream infections (Simon et al., 2008; Van Den Bosch et al., 2024; Dümichen et al., 2012; Handrup et al., 2013; Ince et al., 2014; Lambe et al., 2018). The pooled analysis showed that the cumulative incidence was significantly lower in the taurolidine group than in the control group. The RR of the overall incidence of CRBSI between the two groups was 0.37 (95% CI: 0.23–0.61, I^2^ = 54%, P = 0.0001) (Figure 5).

**FIGURE 5:**
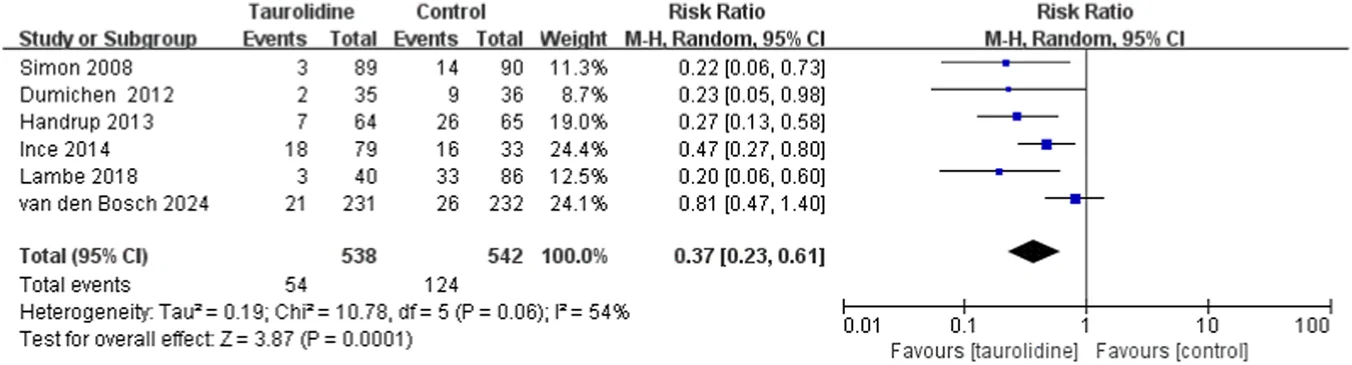
Forest plot of the meta-analysis for cumulative incidence of catheter-related bloodstream infection.

#### 3.3.4 Catheter removal due to infection

Of the 9 included articles, 4 provided the data (Simon et al., 2008; Van Den Bosch et al., 2024; Dümichen et al., 2012; Handrup et al., 2013). The results showed that there was no significant difference between the two groups (RR = 1.25, 95% CI: 0.60–2.62, I^2^ = 12%, P = 0.56) (Figure 6).

**FIGURE 6:**
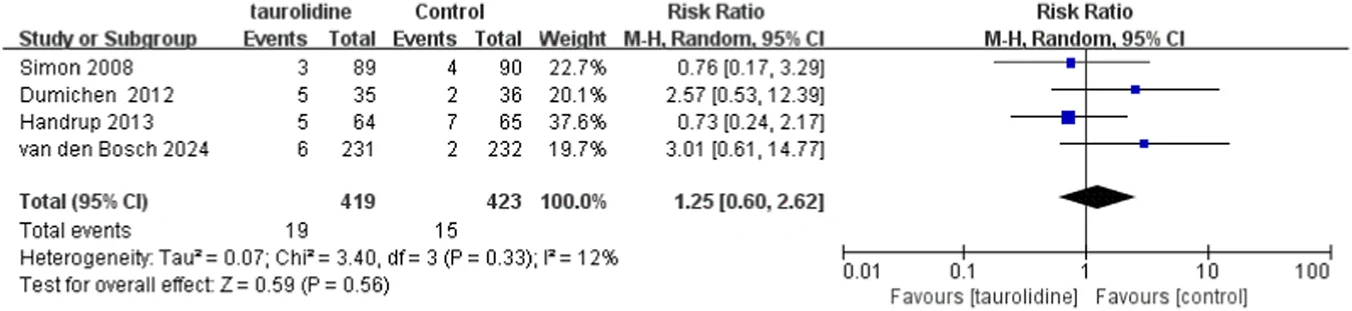
Forest plot of the meta-analysis for catheter removal due to infection.

#### 3.3.5 CVAD-related thrombosis

Of the 9 included articles, 2 provided the data (Van Den Bosch et al., 2024; Dümichen et al., 2012). There was no significant difference in the incidence of thrombosis between the two groups (RR = 0.88, 95% CI: 0.29–2.64, I^2^ = 0%, P = 0.82) (Figure 7).

**FIGURE 7:**
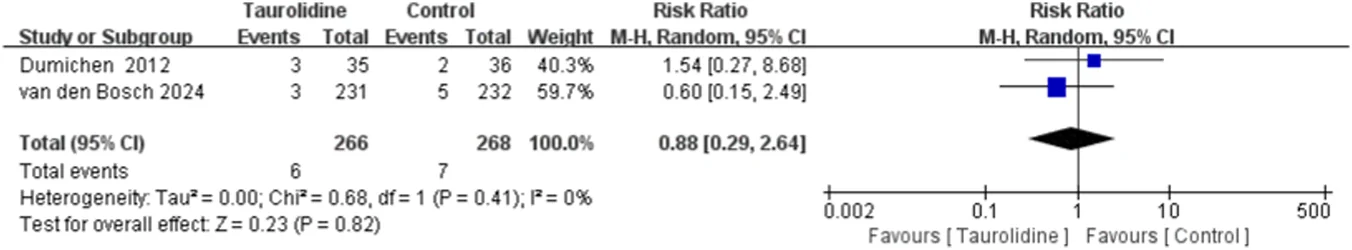
Forest plot of the meta-analysis for CVAD-related thrombosis.

#### 3.3.6 Adverse events

Of the 9 included articles, 4 provided the data (Simon et al., 2008; Van Den Bosch et al., 2024; Dümichen et al., 2012; Handrup et al., 2013). There was no significant difference in the incidence of adverse events between the two groups (RR = 3.93, 95% CI: 0.82–18.80, I^2^ = 64%, P = 0.09). Adverse events reported in the taurolidine-containing lock groups included taste disturbances, nausea and vomiting, and pain (Figure 8).

**FIGURE 8:**
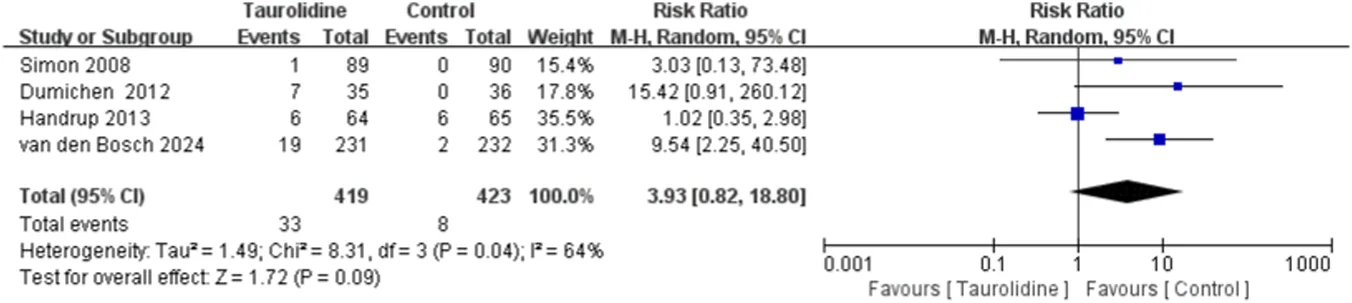
Forest plot of the meta-analysis for adverse events.

## 4 Discussion

### 4.1 General interpretation of the results in the context of other evidence

CRBSI and CLABSI should not be interpreted as equivalent outcomes. CRBSI is intended to identify infections that are clinically or microbiologically attributable to the catheter, whereas CLABSI is a broader surveillance outcome based primarily on the temporal presence of a central line. A CLABSI event may therefore include a bloodstream infection for which the catheter is not the confirmed source. The separate analyses used in the present review reduce outcome misclassification and avoid assuming that the two definitions measure the same underlying event. Accordingly, the CRBSI analysis was considered the principal efficacy analysis, whereas the CLABSI findings provide complementary but less certain evidence.

In the primary analysis restricted to studies reporting CRBSI, taurolidine-containing catheter lock solutions were associated with a significantly lower incidence density of infection than control lock solutions (IRR = 0.23, 95% CI 0.15–0.36) (Simon et al., 2008; Dümichen et al., 2012; Handrup et al., 2013; Hulshof et al., 2017; Lambe et al., 2018). No statistical heterogeneity was detected across the five contributing studies (I^2^ = 0%). Taurolidine-containing lock solutions were associated with an approximately 77% lower incidence rate of CRBSI than control lock solutions. Nevertheless, the absence of statistical heterogeneity should not be interpreted as evidence of complete clinical homogeneity, because the analysis included only five studies and differences remained in patient populations, catheter types, diagnostic criteria, and study designs.

The leave-one-out sensitivity analysis supported the robustness of the primary CRBSI finding. After sequential omission of the five contributing studies, the pooled IRRs ranged from 0.22 (95% CI 0.13–0.37) to 0.25 (95% CI 0.14–0.43), and all confidence intervals remained below 1 (Supplementary Figure S1). Exclusion of Hulshof et al., which had the greatest analytical weight, did not change the direction or statistical significance of the association. These findings indicate that the pooled CRBSI result was not driven by any single study. However, leave-one-out stability cannot address biases shared across studies or differences in populations, catheter-care practices, and lock protocols.

The RCT-only sensitivity analysis produced an effect estimate similar to that of the overall primary analysis (IRR = 0.26, 95% CI 0.11–0.58; I^2^ = 0%) (Supplementary Figure S3), indicating that the observed association was also present when the analysis was restricted to randomized evidence. However, only two RCTs contributed to this analysis, and the confidence interval for Dümichen et al. crossed the null. The RCT-only result therefore supports the direction of the primary finding but does not eliminate the uncertainty arising from the limited randomized evidence.

In the separate secondary analysis of three studies reporting CLABSI, taurolidine was also associated with a lower incidence density (IRR = 0.25, 95% CI 0.08–0.85) (Van Den Bosch et al., 2024; Clark et al., 2019; Chong et al., 2020). However, this result was accompanied by substantial heterogeneity (I^2^ = 90%) and should therefore be interpreted more cautiously. In particular, the effect estimate reported by van den Bosch et al. was closer to the null than those reported by Clark et al. and Chong et al. Differences in baseline infection risk, patient characteristics, catheter types, catheter-care practices, study design, and surveillance definitions may have contributed to the observed inconsistency.

In contrast, the CLABSI finding was sensitive to the omission of individual studies (Supplementary Figure S4). Exclusion of Clark et al. yielded an IRR of 0.40 (95% CI 0.10–1.59), whereas exclusion of Chong et al. yielded an IRR of 0.28 (95% CI 0.03–2.45); both confidence intervals crossed 1. Exclusion of van den Bosch et al. produced a stronger and statistically significant association (IRR = 0.15, 95% CI 0.07–0.32). Although all point estimates remained below 1, the magnitude, precision, and statistical significance of the pooled estimate depended on which study was omitted. Together with the substantial heterogeneity and the inclusion of only three studies, this instability indicates considerable uncertainty regarding the CLABSI effect estimate.

Our findings are broadly consistent with the previous meta-analysis by Sun et al., which included four studies and reported that taurolidine reduced the risk of CRBSI (RR = 0.23, 95% CI 0.13–0.40; I^2^ = 0%). The present review expanded the range of eligible study designs and incorporated additional evidence, including observational studies and the recent randomized trial by van den Bosch et al. In the updated primary CRBSI analysis, the pooled IRR was also 0.23, indicating that the direction and approximate magnitude of the overall association remained consistent with the findings of Sun et al. (2020). However, direct numerical comparison should be made cautiously because Sun et al. reported a patient-level risk ratio, whereas the present primary analysis used an incidence rate ratio based on catheter-days.

The updated evidence provides a more nuanced interpretation than the earlier review. Although the primary CRBSI analysis remained statistically significant and internally consistent, the CLABSI analysis showed considerable heterogeneity, and the most recent randomized trial reported a substantially more conservative effect estimate. Therefore, the additional evidence did not reverse the overall conclusion that taurolidine may reduce catheter-related bloodstream infections, but it indicates that the magnitude of benefit may not be uniform across clinical settings and that the certainty of the evidence remains limited.

Two included studies also reported the use of prophylactic antibiotics during the study period (Simon et al., 2008; Dümichen et al., 2012). Such co-interventions may alter baseline infection risk and modify the observed relative effect of the lock regimen. Because the indications, timing, duration, and between-group distribution of prophylactic antibiotic exposure were not consistently reported, its independent contribution could not be separated from that of the catheter lock intervention. The direction and magnitude of any resulting confounding therefore remain uncertain, particularly in the observational studies.

Four studies reported catheter removal due to infection. In this meta-analysis, taurolidine lock solution did not significantly reduce catheter removal compared with the control group (Simon et al., 2008; Van Den Bosch et al., 2024; Dümichen et al., 2012; Handrup et al., 2013). This finding should be interpreted cautiously, as catheter removal is a clinical management outcome rather than a direct adverse effect of taurolidine. The decision to remove a CVAD may be influenced by infection severity, local management protocols, catheter function, and institutional removal criteria, which may explain the lack of a significant difference between groups. Common causes of CVAD removal include bacterial colonization at the catheter exit, obstruction of the catheter lumen, and poor local inflammation control. Although bacterial colonization at the exit of the catheter does not lead to systemic infection, preventive removal is often used in clinical practice to prevent the spread of infection. In individual studies, some patients were suspected of infection due to fever or elevated inflammatory markers, and although not confirmed to be associated with taurolidine, they were still extubated due to clinical caution.

CVAD-related thrombosis was reported in two studies, both of which were RCTs; therefore, this outcome represents an RCT-only pooled analysis (Van Den Bosch et al., 2024; Dümichen et al., 2012). Although no statistically significant between-group difference was observed (RR = 0.88, 95% CI 0.29–2.64), only two trials contributed to the analysis, and the confidence interval was wide. Therefore, this finding should not be interpreted as evidence of equivalent thrombosis risk between the two groups. The two contributing trials evaluated different taurolidine-containing formulations: one used taurolidine-citrate without heparin in the intervention arm, whereas the other used a taurolidine-citrate-heparin formulation. Because heparin may independently influence catheter patency and thrombosis, the pooled estimate cannot distinguish the effect of taurolidine from the potential contribution of heparin and should therefore be regarded as exploratory. The formation of thrombosis may be related to mechanical damage to the vascular endothelium caused by long-term catheter indwelling, which leads to activation of the local coagulation system and deposition of fibrin (Citla Sridhar et al., 2020). In addition, the hypercoagulable state of patients also plays a key role in thrombosis, such as children with tumors, critically ill patients or patients receiving long-term parenteral nutrition often have coagulation dysfunction, platelet activation and fibrinogen elevation, which significantly increase the risk of thrombosis (Revel-Vilk et al., 2010). Management of CVAD-related thrombosis generally depends on clinical severity and may include anticoagulation, supportive treatment, and catheter removal when clinically indicated.

Four included studies provided adverse-event data (Simon et al., 2008; Van Den Bosch et al., 2024; Dümichen et al., 2012; Handrup et al., 2013). The pooled estimate did not reach statistical significance (RR = 3.93, 95% CI 0.82–18.80; I^2^ = 64%; P = 0.09); however, the point estimate and wide confidence interval indicate substantial uncertainty and should not be interpreted as evidence of comparable safety. Adverse events reported in the taurolidine-containing lock groups included oral dysgeusia or dysesthesia, nausea and vomiting, and transient pain. The included studies did not consistently assess the relationship between these events and the individual components of the lock formulations. Consequently, the respective contributions of taurolidine, citrate, heparin, stabilizers, underlying disease, or concomitant treatment could not be determined, and the reported events should not be regarded as adverse effects caused specifically by taurolidine. Moreover, the included studies did not systematically evaluate antimicrobial resistance and provided limited long-term microbiological surveillance and safety follow-up. Overall, the current evidence is insufficient to establish the comparative safety of taurolidine-containing lock solutions or to determine their long-term effects on antimicrobial resistance and toxicity. Future adequately powered trials should use standardized safety definitions and incorporate long-term adverse-event monitoring and microbiological surveillance.

### 4.2 Limitations of the evidence included in the review

Several limitations of the available evidence should be acknowledged. First, only nine studies were included, of which three were randomized controlled trials and six were retrospective cohort studies. The predominance of observational evidence increases susceptibility to residual confounding, selection bias, and temporal changes in catheter-care practices. Several studies also had small sample sizes, resulting in limited statistical power and imprecise estimates. In addition, follow-up was generally less than 1 year, restricting the assessment of long-term efficacy, delayed adverse events, antimicrobial resistance, and catheter-related complications.

Second, substantial clinical diversity remained across the included studies. The populations included children with oncological or hematological diseases, intestinal failure requiring home parenteral nutrition, and critical illness. Catheter types, baseline infection risks, comparator regimens, catheter-care practices, lock volumes, dwell times, administration frequencies, and treatment durations also differed. In addition, the taurolidine-containing formulations were not uniform: five studies used 1.35% taurolidine with 4% sodium citrate; one used 1.34% taurolidine with 4% sodium citrate; two used 1.35% taurolidine with 4% sodium citrate and 100 IU/mL heparin; and one used 2% taurolidine alone. Although these formulations were pooled to estimate the overall association of taurolidine-containing lock regimens as a class, they should not be considered clinically interchangeable. In particular, heparin may independently affect catheter patency, thrombosis, and other catheter-related outcomes, and its contribution could not be separated from that of taurolidine. The small number of studies within each formulation category precluded reliable formulation-specific analyses. Therefore, even though no statistical heterogeneity was detected in the primary CRBSI analysis, the pooled estimate should not be assumed to apply equally to every formulation, pediatric population, or lock protocol.

Third, infection definitions were not uniform. Separating CRBSI from CLABSI reduced one important source of outcome heterogeneity, but the diagnostic criteria used within each category were not completely consistent across studies. Moreover, only five studies contributed to the CRBSI incidence-density analysis and three to the CLABSI analysis. These small numbers limited the statistical power of subgroup analyses and made a reliable meta-regression inappropriate. The substantial heterogeneity in the CLABSI analysis and the moderate heterogeneity in the cumulative-incidence and adverse-event analyses indicate that important clinical and methodological differences remain unexplained.

Fourth, the evidence for safety-related outcomes was sparse. Only two studies reported CVAD-related thrombosis, four reported adverse events, and few studies conducted systematic or long-term microbiological surveillance. The wide confidence intervals surrounding the thrombosis and adverse-event estimates indicate substantial imprecision. Therefore, the absence of statistically significant differences should not be interpreted as evidence of equivalence or absence of harm. Overall, the small evidence base, predominance of observational studies, clinical and methodological diversity, incomplete reporting of lock protocols, and limited safety data reduce confidence in the precision and generalizability of the findings.

### 4.3 Limitations of the review processes used

Although four electronic databases and the reference lists of relevant publications were searched, no separate searches of dedicated grey literature sources, trial registry websites, or conference websites were conducted. Relevant records from these sources were screened when retrieved through the database searches, but unpublished studies and incompletely indexed reports may still have been missed.

The review relied on aggregate data and the infection definitions reported by the original investigators; individual infection episodes could not always be independently reclassified using uniform diagnostic criteria. Incomplete reporting of lock composition, exposure duration, catheter-care procedures, and concomitant prophylactic antibiotics also restricted further analyses of potential effect modifiers.

The use of the Newcastle–Ottawa Scale to assess the non-randomized cohort studies also has limitations. Although the NOS provides a structured assessment of selection, comparability, and outcome ascertainment, its star-based system may not fully capture bias arising from residual confounding, deviations from intended interventions, selection into the analysis, or selective reporting. Moreover, judgments regarding comparability depend on the confounding factors reported and controlled for in the original studies. Item-level assessments were therefore reported, and the total NOS score was not interpreted as a definitive overall judgment of study quality. Nevertheless, the risk of bias in the observational evidence may not have been fully characterized.

The CRBSI funnel plot appeared broadly balanced, whereas the CLABSI funnel plot showed a more uneven distribution of study estimates (Supplementary Figures S2, S5). However, the plots contained only five and three studies, respectively, making visual assessment highly uncertain. The apparent balance of the CRBSI plot cannot exclude publication bias, while the apparent asymmetry of the CLABSI plot cannot establish its presence. In the CLABSI analysis, substantial heterogeneity, differences in study size, and variation in study populations and methods provide plausible alternative explanations for the observed pattern. Therefore, both funnel plots were interpreted as exploratory, and the possibility of unpublished or incompletely indexed evidence remains unresolved.

The presentation of results primarily as relative effect estimates limits the assessment of the absolute clinical benefit. A large relative reduction does not necessarily translate into a large absolute reduction, which depends on the baseline infection risk or rate and the observation period. The pooled relative effects should therefore not be interpreted as indicating a uniformly large clinical benefit across settings.

### 4.4 Implications of the results for practice, policy, and future research

The available evidence suggests that taurolidine-containing lock regimens may reduce bloodstream infections in some pediatric patients requiring long-term central venous access. However, the limited randomized evidence, variation in lock formulations and protocols, substantial heterogeneity in the CLABSI analysis, inconsistent infection definitions, and insufficient safety data do not support universal or routine implementation or the designation of these regimens as a preferred preventive strategy. Where a taurolidine-containing regimen is considered for an individual patient, the decision should account for baseline infection risk, previous catheter infections, catheter type, the specific formulation and protocol, concomitant preventive measures, and the uncertainty surrounding long-term safety.

Future studies should be adequately powered, multicenter randomized controlled trials conducted in clearly defined pediatric populations. Investigators should report the exact taurolidine concentration and formulation, citrate or heparin content, lock volume, dwell time, administration frequency, catheter type, duration of treatment, comparator protocol, and concomitant antimicrobial prophylaxis. CRBSI and CLABSI should be prospectively defined and reported separately, and studies should include standardized assessments of adverse events, thrombosis, catheter removal, and microbiological resistance over sufficiently long follow-up periods. Direct comparisons of different taurolidine-containing formulations and formal cost-effectiveness analyses would also help identify the most appropriate regimen and target population. If clinically meaningful efficacy and an acceptable safety profile are confirmed by robust large-scale trials, taurolidine-containing lock regimens could provide substantial preventive and clinical benefits in pediatric catheter care. Conversely, high-quality evidence demonstrating no clinically meaningful benefit would remain valuable by discouraging ineffective interventions and supporting more efficient allocation of healthcare resources.

## 5 Conclusion

In conclusion, taurolidine-containing lock solutions may reduce catheter-related bloodstream infections in selected high-risk pediatric patients requiring long-term central venous access. However, the available evidence is insufficient to establish their comparative safety. The findings should be interpreted cautiously because of variation in lock protocols, mixed study designs, inconsistent infection definitions, and limited safety data. Further multicenter randomized controlled trials with adequate long-term safety monitoring are needed before routine implementation can be recommended.

## Data Availability

The datasets generated/analyzed for this study can be found in Figshare: https://doi.org/10.6084/m9.figshare.30565799.
