# Supplementary material for: The efficacy of taurolidine lock solution in preventing catheter-related bloodstream infections in pediatrics: a systematic review and meta-analysis
Source: Front Pharmacol. 2026 Oct 21;17:1938862. doi: 10.3389/fphar.2026.1938862 (PMC13732722; doi:10.3389/fphar.2026.1938862)
Supplement: Supplementary file 1 [file Table1.docx]

| Pubmed | | |
| --- | --- | --- |
| No. | Query | Results |
| #1 | ((((((((((((((((((((((((((((ethanol lock[Title/Abstract]) OR (citrate lock[Title/Abstract])) OR (hydrochloric acid lock[Title/Abstract])) OR (taurolidine lock[Title/Abstract])) OR (taurolock[Title/Abstract])) OR (urokinase[Title/Abstract])) OR (lock solution[Title/Abstract])) OR (flush solution[Title/Abstract])) OR (flush[Title/Abstract])) OR (flushing[Title/Abstract])) OR (antibiotic[Title/Abstract])) OR (antibiotics[Title/Abstract])) OR (lock[Title/Abstract])) OR (locks[Title/Abstract])) OR (locking[Title/Abstract])) OR (lock therapy[Title/Abstract])) OR (lock treatment[Title/Abstract])) OR (lock regimen[Title/Abstract])) OR (lock technique[Title/Abstract])) OR (antibiotic lock[Title/Abstract])) OR (antibiotic locks[Title/Abstract])) OR (antibiotic-lock[Title/Abstract])) OR (antibiotic-locks[Title/Abstract])) OR (antibiotic locking[Title/Abstract])) OR (antibiotic-locking[Title/Abstract])) OR (antibiotic lock treatment[Title/Abstract])) OR (ALT[Title/Abstract])) OR (Antimicrobial lock technique[Title/Abstract])) OR (catheter lock[Title/Abstract]) | 647,804 |
| #2 | (((((((((((((((((((((((((((((((((((((((((((central venous catheters[MeSH Terms]) OR (Catheter, Central Venous[Title/Abstract])) OR (Catheters, Central Venous[Title/Abstract])) OR (Venous Catheter, Central[Title/Abstract])) OR (Venous Catheters, Central[Title/Abstract])) OR (Central Venous Catheter[Title/Abstract])) OR (Central vascular access device[Title/Abstract])) OR (CVCs[Title/Abstract])) OR (catheter-related infections[Title/Abstract])) OR (catheter related complications[Title/Abstract])) OR (broviac[Title/Abstract])) OR (port-a-cath[Title/Abstract])) OR (port a cath[Title/Abstract])) OR (implantable port[Title/Abstract])) OR (implantable ports[Title/Abstract])) OR (implantable catheter[Title/Abstract])) OR (implantable catheters[Title/Abstract])) OR (totally implantable access port[Title/Abstract])) OR (totally implantable access ports[Title/Abstract])) OR (Implantable Catheters[Title/Abstract])) OR (Implantable Catheter[Title/Abstract])) OR (Vascular Access Ports[Title/Abstract])) OR (Vascular Access Port[Title/Abstract])) OR (TIAP[Title/Abstract])) OR (TIAPs[Title/Abstract])) OR (hickman[Title/Abstract])) OR (catheterization central venous[Title/Abstract])) OR (tunnelled central venous catheter[Title/Abstract])) OR (TCVC[Title/Abstract])) OR (catheterization central venous[Title/Abstract])) OR (peripherally inserted central Catheter[Title/Abstract])) OR (picc[Title/Abstract])) OR (central venous line[Title/Abstract])) OR (central venous device[Title/Abstract])) OR (central venous access device[Title/Abstract])) OR (CVAD[Title/Abstract])) OR (CVC[Title/Abstract])) OR (Indwelling Catheter[Title/Abstract])) OR (Indwelling Catheters[Title/Abstract])) OR (In Dwelling Catheters[Title/Abstract])) OR (In Dwelling Catheters[Title/Abstract])) OR (In-Dwelling Catheter[Title/Abstract])) OR (Venous Reservoirs[Title/Abstract])) OR (Venous Reservoir[Title/Abstract]) | 29,377 |
| #3 | ((((((((((((((((((((((((((((((((((((((((((((((((((((((((((Catheter-Related Infections[MeSH Terms]) OR (Catheter-Related Infection[Title/Abstract])) OR (Catheter Related Infections[Title/Abstract])) OR (Infection, Catheter-Related[Title/Abstract])) OR (Infections, Catheter-Related[Title/Abstract])) OR (Catheter-Associated Infections[Title/Abstract])) OR (Catheter-Associated Infection[Title/Abstract])) OR (Catheter Associated Infections[Title/Abstract])) OR (Infection, Catheter-Associated[Title/Abstract])) OR (Infections, Catheter-Associated[Title/Abstract])) OR (Catheter Related Infection[Title/Abstract])) OR (Infection, Catheter Related[Title/Abstract])) OR (Infections, Catheter Related[Title/Abstract])) OR (Related Infection, Catheter[Title/Abstract])) OR (Related Infections, Catheter[Title/Abstract])) OR (Catheter Associated Infection[Title/Abstract])) OR (Associated Infection, Catheter[Title/Abstract])) OR (Associated Infections, Catheter[Title/Abstract])) OR (Infection, Catheter Associated[Title/Abstract])) OR (Infections, Catheter Associated[Title/Abstract])) OR (catheter-related bloodstream infection[Title/Abstract])) OR (catheter-related bloodstream infections[Title/Abstract])) OR (CRBSI[Title/Abstract])) OR (CRBSIs[Title/Abstract])) OR (catheter-related bacteremia[Title/Abstract])) OR (catheter-related bacteremias[Title/Abstract])) OR (catheter-related bacteraemia[Title/Abstract])) OR (catheter-related bacteraemias[Title/Abstract])) OR (BSI[Title/Abstract])) OR (CABSI[Title/Abstract])) OR (CABSIs[Title/Abstract])) OR (catheter associated bloodstream infection[Title/Abstract])) OR (catheter-associated bloodstream infections[Title/Abstract])) OR (catheter related complications[Title/Abstract])) OR (Sepsis[Title/Abstract])) OR (bacteremia[Title/Abstract])) OR (bacteremias[Title/Abstract])) OR (bacteraemia[Title/Abstract])) OR (infection[Title/Abstract])) OR (infections[Title/Abstract])) OR (line infection[Title/Abstract])) OR (line infections[Title/Abstract])) OR (bloodstream infection[Title/Abstract])) OR (bloodstream infections[Title/Abstract])) OR (infectious diseases[Title/Abstract])) OR (Pyemia[Title/Abstract])) OR (Pyemias[Title/Abstract])) OR (Pyohemia[Title/Abstract])) OR (Pyohemias[Title/Abstract])) OR (Pyaemia[Title/Abstract])) OR (Pyaemias[Title/Abstract])) OR (Septicemia[Title/Abstract])) OR (Septicemias[Title/Abstract])) OR (Septicaemia[Title/Abstract])) OR (Septicaemias[Title/Abstract])) OR (Severe Sepsis[Title/Abstract])) OR (CVAD-associated grampositive infections[Title/Abstract])) OR (central line–associated blood stream infection[Title/Abstract])) OR (Contamination[Title/Abstract]) | 2,423,867 |
| #4 | (((((((((((((((((((((((((((((((((((((((((((((Infant[MeSH Terms]) OR (Infan[Title/Abstract])) OR (Infants[Title/Abstract])) OR (premature infants[Title/Abstract])) OR (Infants, Newborn[Title/Abstract])) OR (Newborn Infant[Title/Abstract])) OR (Newborn Infants[Title/Abstract])) OR (Neonate[Title/Abstract])) OR (Neonates[Title/Abstract])) OR (Newborns[Title/Abstract])) OR (Newborn[Title/Abstract])) OR (babies[Title/Abstract])) OR (baby[Title/Abstract])) OR (Neonat[Title/Abstract])) OR (Perinat[Title/Abstract])) OR (postnat[Title/Abstract])) OR (Schoolchild[Title/Abstract])) OR (school child[Title/Abstract])) OR (Child[MeSH Terms])) OR (Children[Title/Abstract])) OR (Preschool Child[Title/Abstract])) OR (Children, Preschool[Title/Abstract])) OR (Preschool Children[Title/Abstract])) OR (Kid[Title/Abstract])) OR (kids[Title/Abstract])) OR (pediatrics[MeSH Terms])) OR (pediatric[Title/Abstract])) OR (Pediatric patients[Title/Abstract])) OR (adoles[Title/Abstract])) OR (Adolescent[MeSH Terms])) OR (Adolescents[Title/Abstract])) OR (Adolescence[Title/Abstract])) OR (Adolescents, Female[Title/Abstract])) OR (Adolescent, Female[Title/Abstract])) OR (Female Adolescent[Title/Abstract])) OR (Female Adolescents[Title/Abstract])) OR (Adolescents, Male[Title/Abstract])) OR (Adolescent, Male[Title/Abstract])) OR (Male Adolescent[Title/Abstract])) OR (Male Adolescents[Title/Abstract])) OR (Youth[Title/Abstract])) OR (Youths[Title/Abstract])) OR (Teens[Title/Abstract])) OR (Teen[Title/Abstract])) OR (Teenagers[Title/Abstract])) OR (Teenager[Title/Abstract]) | 5,000,155 |
| #5 | (((randomized controlled trial [pt] OR controlled clinical trial [pt] OR randomized [tiab] OR placebo [tiab] OR clinical trials as topic [mesh: noexp] OR randomly [tiab] OR trial [ti]) NOT (animals [mh] NOT humans [mh])) OR (Prospective[Title/Abstract])) OR (Retrospective[Title/Abstract]) | 3,457,677 |
| #6 | #1 AND #2 AND #3 AND #4 AND #5 | 432 |

| Cochrane library | | |
| --- | --- | --- |
| No. | Query | Results |
| #1 | (ethanol lock):ti,ab,kw OR (citrate lock):ti,ab,kw OR (hydrochloric acid lock):ti,ab,kw OR (taurolidine lock):ti,ab,kw OR (taurolock):ti,ab,kw OR (urokinase):ti,ab,kw OR (lock solution):ti,ab,kw OR (flush solution):ti,ab,kw OR (flush):ti,ab,kw OR (flushing):ti,ab,kw OR (antibiotic):ti,ab,kw OR (antibiotics):ti,ab,kw OR (lock):ti,ab,kw OR (locks):ti,ab,kw OR (locking):ti,ab,kw OR (lock therapy):ti,ab,kw OR (lock treatment):ti,ab,kw OR (lock regimen):ti,ab,kw OR (lock technique):ti,ab,kw OR (antibiotic lock):ti,ab,kw OR (antibiotic locks):ti,ab,kw OR (antibiotic-lock):ti,ab,kw OR (antibiotic-locks):ti,ab,kw OR (antibiotic locking):ti,ab,kw OR (antibiotic-locking):ti,ab,kw OR (antibiotic lock treatment):ti,ab,kw OR (ALT):ti,ab,kw OR (Antimicrobial lock technique):ti,ab,kw OR (catheter lock):ti,ab,kw | 67,344 |
| #2 | MeSH descriptor: [Central Venous Catheters] explode all trees | 10,553 |
|  | (Catheter, Central Venous):ti,ab,kw OR (Catheters, Central Venous):ti,ab,kw OR (Venous Catheter, Central):ti,ab,kw OR (Venous Catheters, Central):ti,ab,kw OR (Central Venous Catheter):ti,ab,kw OR (Central vascular access device):ti,ab,kw OR (CVCs):ti,ab,kw OR (catheter-related infections):ti,ab,kw OR (catheter related complications):ti,ab,kw OR (broviac):ti,ab,kw OR (port-a-cath):ti,ab,kw OR (port a cath):ti,ab,kw OR (implantable port):ti,ab,kw OR (implantable ports):ti,ab,kw OR (implantable catheter):ti,ab,kw OR (implantable catheters):ti,ab,kw OR (totally implantable access port):ti,ab,kw OR (totally implantable access ports):ti,ab,kw OR (Implantable Catheters):ti,ab,kw OR (Implantable Catheter):ti,ab,kw OR (Vascular Access Ports):ti,ab,kw OR (Vascular Access Port):ti,ab,kw OR (TIAP):ti,ab,kw OR (TIAPs):ti,ab,kw OR (hickman):ti,ab,kw OR (catheterization central venous):ti,ab,kw OR (tunnelled central venous catheter):ti,ab,kw OR (TCVC):ti,ab,kw OR (catheterization central venous):ti,ab,kw OR (peripherally inserted central Catheter):ti,ab,kw OR (picc):ti,ab,kw OR (central venous line):ti,ab,kw OR (central venous device):ti,ab,kw OR (central venous access device):ti,ab,kw OR (CVAD):ti,ab,kw OR (CVC):ti,ab,kw OR (Indwelling Catheter):ti,ab,kw OR (Indwelling Catheters):ti,ab,kw OR (In Dwelling Catheters):ti,ab,kw OR (In Dwelling Catheters):ti,ab,kw OR (In-Dwelling Catheter):ti,ab,kw OR (Venous Reservoirs):ti,ab,kw OR (Venous Reservoir):ti,ab,kw |  |
| #3 | MeSH descriptor: [Catheter-Related Infections] explode all trees | 182,826 |
|  | (Catheter-Related Infection):ti,ab,kw OR (Catheter Related Infections):ti,ab,kw OR (Infection, Catheter-Related):ti,ab,kw OR (Infections, Catheter-Related):ti,ab,kw OR (Catheter-Associated Infections):ti,ab,kw OR (Catheter-Associated Infection):ti,ab,kw OR (Catheter Associated Infections):ti,ab,kw OR (Infection, Catheter-Associated):ti,ab,kw OR (Infections, Catheter-Associated):ti,ab,kw OR (Catheter Related Infection):ti,ab,kw OR (Infection, Catheter Related):ti,ab,kw OR (Infections, Catheter Related):ti,ab,kw OR (Related Infection, Catheter):ti,ab,kw OR (Related Infections, Catheter):ti,ab,kw OR (Catheter Associated Infection):ti,ab,kw OR (Associated Infection, Catheter):ti,ab,kw OR (Associated Infections, Catheter):ti,ab,kw OR (Infection, Catheter Associated):ti,ab,kw OR (Infections, Catheter Associated):ti,ab,kw OR (catheter-related bloodstream infection):ti,ab,kw OR (catheter-related bloodstream infections):ti,ab,kw OR (CRBSI):ti,ab,kw OR (CRBSIs):ti,ab,kw OR (catheter-related bacteremia):ti,ab,kw OR (catheter-related bacteremias):ti,ab,kw OR (catheter-related bacteraemia):ti,ab,kw OR (catheter-related bacteraemias):ti,ab,kw OR (BSI):ti,ab,kw OR (CABSI):ti,ab,kw OR (CABSIs):ti,ab,kw OR (catheter associated bloodstream infection):ti,ab,kw OR (catheter-associated bloodstream infections):ti,ab,kw OR (catheter related complications):ti,ab,kw OR (Sepsis):ti,ab,kw OR (bacteremia):ti,ab,kw OR (bacteremias):ti,ab,kw OR (bacteraemia):ti,ab,kw OR (infection):ti,ab,kw OR (infections):ti,ab,kw OR (line infection):ti,ab,kw OR (line infections):ti,ab,kw OR (bloodstream infection):ti,ab,kw OR (bloodstream infections):ti,ab,kw OR (infectious diseases):ti,ab,kw OR (Pyemia):ti,ab,kw OR (Pyemias):ti,ab,kw OR (Pyohemia):ti,ab,kw OR (Pyohemias):ti,ab,kw OR (Pyaemia):ti,ab,kw OR (Pyaemias):ti,ab,kw OR (Septicemia):ti,ab,kw OR (Septicemias):ti,ab,kw OR (Septicaemia):ti,ab,kw OR (Septicaemias):ti,ab,kw OR (Severe Sepsis):ti,ab,kw OR (CVAD-associated grampositive infections):ti,ab,kw OR (central line–associated blood stream infection):ti,ab,kw OR (Contamination):ti,ab,kw |  |
| #4 | MeSH descriptor：[Adolescent] explode all trees | 393,276 |
|  | MeSH descriptor： [infant, Newborn] explode all trees |  |
|  | MeSH descriptor：[pediatrics] explode all trees |  |
|  | MeSH descriptor：[Child] explode all trees |  |
|  | (Infan):ti,ab,kw OR (Infants):ti,ab,kw OR (premature infants):ti,ab,kw OR (Infants, Newborn):ti,ab,kw OR (Newborn Infant):ti,ab,kw OR (Newborn Infants):ti,ab,kw OR (Neonate):ti,ab,kw OR (Neonates):ti,ab,kw OR (Newborns):ti,ab,kw OR (Newborn):ti,ab,kw OR (babies):ti,ab,kw OR (baby):ti,ab,kw OR (Neonat):ti,ab,kw OR (Perinat):ti,ab,kw OR (postnat):ti,ab,kw OR (Schoolchild):ti,ab,kw OR (school child):ti,ab,kw OR (Children):ti,ab,kw OR (Preschool Child):ti,ab,kw OR (Children, Preschool):ti,ab,kw OR (Preschool Children):ti,ab,kw OR (Kid):ti,ab,kw OR (kids):ti,ab,kw OR (pediatric):ti,ab,kw OR (Pediatric patients):ti,ab,kw OR (adoles):ti,ab,kw OR (Adolescents):ti,ab,kw OR (Adolescence):ti,ab,kw OR (Adolescents, Female):ti,ab,kw OR (Adolescent, Female):ti,ab,kw OR (Female Adolescent):ti,ab,kw OR (Female Adolescents):ti,ab,kw OR (Adolescents, Male):ti,ab,kw OR (Adolescent, Male):ti,ab,kw OR (Male Adolescent):ti,ab,kw OR (Male Adolescents):ti,ab,kw OR (Youth):ti,ab,kw OR (Youths):ti,ab,kw OR (Teens):ti,ab,kw OR (Teen):ti,ab,kw OR (Teenagers):ti,ab,kw OR (Teenager):ti,ab,kw |  |
| #5 | (randomized controlled trial OR controlled clinical trial OR Randomized OR placebo OR clinical trials as topic OR randomly OR Trial OR Prospective OR Retrospective):ti,ab,kw | 1,787,237 |
| #6 | #1 AND #2 AND #3 AND #4 AND #5 | 281 |

| Embase | | |
| --- | --- | --- |
| No. | Query | Results |
| #1 | (ethanol lock):ti,ab,kw OR (citrate lock):ti,ab,kw OR (hydrochloric acid lock):ti,ab,kw OR (taurolidine lock):ti,ab,kw OR (taurolock):ti,ab,kw OR (urokinase):ti,ab,kw OR (lock solution):ti,ab,kw OR (flush solution):ti,ab,kw OR (flush):ti,ab,kw OR (flushing):ti,ab,kw OR (antibiotic):ti,ab,kw OR (antibiotics):ti,ab,kw OR (lock):ti,ab,kw OR (locks):ti,ab,kw OR (locking):ti,ab,kw OR (lock therapy):ti,ab,kw OR (lock treatment):ti,ab,kw OR (lock regimen):ti,ab,kw OR (lock technique):ti,ab,kw OR (antibiotic lock):ti,ab,kw OR (antibiotic locks):ti,ab,kw OR (antibiotic-lock):ti,ab,kw OR (antibiotic-locks):ti,ab,kw OR (antibiotic locking):ti,ab,kw OR (antibiotic-locking):ti,ab,kw OR (antibiotic lock treatment):ti,ab,kw OR (ALT):ti,ab,kw OR (Antimicrobial lock technique):ti,ab,kw OR (catheter lock):ti,ab,kw | 928,612 |
| #2 | 'central venous catheter'/exp OR 'central venous catheter' | 70,715 |
|  | 'catheter, central venous':kw,ti,ab OR 'catheters, central venous':kw,ti,ab OR 'venous catheter, central':kw,ti,ab OR 'venous catheters, central':kw,ti,ab OR 'central venous catheter':kw,ti,ab OR 'central vascular access device':kw,ti,ab OR 'cvcs':kw,ti,ab OR 'catheter-related infections':kw,ti,ab OR 'catheter related complications':kw,ti,ab OR 'broviac':kw,ti,ab OR 'port-a-cath':kw,ti,ab OR 'port a cath':kw,ti,ab OR 'implantable port':kw,ti,ab OR 'implantable ports':kw,ti,ab OR 'totally implantable access port':kw,ti,ab OR 'totally implantable access ports':kw,ti,ab OR 'implantable catheters':kw,ti,ab OR 'implantable catheter':kw,ti,ab OR 'vascular access ports':kw,ti,ab OR 'vascular access port':kw,ti,ab OR 'tiap':kw,ti,ab OR 'tiaps':kw,ti,ab OR 'hickman':kw,ti,ab OR 'tunnelled central venous catheter':kw,ti,ab OR 'tcvc':kw,ti,ab OR 'catheterization central venous':kw,ti,ab OR 'peripherally inserted central catheter':kw,ti,ab OR 'picc':kw,ti,ab OR 'central venous line':kw,ti,ab OR 'central venous device':kw,ti,ab OR 'central venous access device':kw,ti,ab OR 'cvad':kw,ti,ab OR 'cvc':kw,ti,ab OR 'indwelling catheter':kw,ti,ab OR 'indwelling catheters':kw,ti,ab OR 'in dwelling catheters':kw,ti,ab OR 'in-dwelling catheter':kw,ti,ab OR 'venous reservoirs':kw,ti,ab OR 'venous reservoir':kw,ti,ab |  |
| #3 | 'catheter infection'/exp OR 'catheter infection' | 3,253,043 |
|  | 'catheter-related infections':kw,ti,ab OR 'catheter-related infection':kw,ti,ab OR 'catheter related infections':kw,ti,ab OR 'infection, catheter-related':kw,ti,ab OR 'infections, catheter-related':kw,ti,ab OR 'catheter-associated infections':kw,ti,ab OR 'catheter-associated infection':kw,ti,ab OR 'catheter associated infections':kw,ti,ab OR 'infection, catheter-associated':kw,ti,ab OR 'infections, catheter-associated':kw,ti,ab OR 'catheter related infection':kw,ti,ab OR 'infection, catheter related':kw,ti,ab OR 'infections, catheter related':kw,ti,ab OR 'related infection, catheter':kw,ti,ab OR 'related infections, catheter':kw,ti,ab OR 'catheter associated infection':kw,ti,ab OR 'associated infection, catheter':kw,ti,ab OR 'associated infections, catheter':kw,ti,ab OR 'infection, catheter associated':kw,ti,ab OR 'infections, catheter associated':kw,ti,ab OR 'catheter-related bloodstream infection':kw,ti,ab OR 'catheter-related bloodstream infections':kw,ti,ab OR 'crbsi':kw,ti,ab OR 'crbsis':kw,ti,ab OR 'catheter-related bacteremia':kw,ti,ab OR 'catheter-related bacteremias':kw,ti,ab OR 'catheter-related bacteraemia':kw,ti,ab OR 'catheter-related bacteraemias':kw,ti,ab OR 'bsi':kw,ti,ab OR 'cabsi':kw,ti,ab OR 'cabsis':kw,ti,ab OR 'catheter associated bloodstream infection':kw,ti,ab OR 'catheter-associated bloodstream infections':kw,ti,ab OR 'catheter related complications':kw,ti,ab OR 'sepsis':kw,ti,ab OR 'bacteremia':kw,ti,ab OR 'bacteremias':kw,ti,ab OR 'bacteraemia':kw,ti,ab OR 'infection':kw,ti,ab OR 'infections':kw,ti,ab OR 'line infection':kw,ti,ab OR 'line infections':kw,ti,ab OR 'bloodstream infection':kw,ti,ab OR 'bloodstream infections':kw,ti,ab OR 'infectious diseases':kw,ti,ab OR 'pyemia':kw,ti,ab OR 'pyemias':kw,ti,ab OR 'pyohemia':kw,ti,ab OR 'pyohemias':kw,ti,ab OR 'pyaemia':kw,ti,ab OR 'pyaemias':kw,ti,ab OR 'septicemia':kw,ti,ab OR 'septicemias':kw,ti,ab OR 'septicaemia':kw,ti,ab OR 'septicaemias':kw,ti,ab OR 'severe sepsis':kw,ti,ab OR 'cvad-associated grampositive infections':kw,ti,ab OR 'central line–associated blood stream infection':kw,ti,ab OR 'contamination':kw,ti,ab |  |
| #4 | 'adolescent'/exp OR 'adolescent' | 6,545,644 |
|  | 'infant'/exp OR 'infant' |  |
|  | 'newborn'/exp OR 'newborn' |  |
|  | 'preschool child'/exp OR 'preschool child' |  |
|  | 'child'/exp OR 'child' |  |
|  | 'pediatrics'/exp OR 'pediatrics' |  |
|  | 'infan':kw,ti,ab OR 'infants':kw,ti,ab OR 'premature infants':kw,ti,ab OR 'infants, newborn':kw,ti,ab OR 'newborn infant':kw,ti,ab OR 'newborn infants':kw,ti,ab OR 'neonate':kw,ti,ab OR 'neonates':kw,ti,ab OR 'newborns':kw,ti,ab OR 'newborn':kw,ti,ab OR 'babies':kw,ti,ab OR 'baby':kw,ti,ab OR 'neonat':kw,ti,ab OR 'perinat':kw,ti,ab OR 'postnat':kw,ti,ab OR 'schoolchild':kw,ti,ab OR 'school child':kw,ti,ab OR 'pediatric':kw,ti,ab OR 'pediatric patients':kw,ti,ab OR 'adoles':kw,ti,ab OR 'adolescents':kw,ti,ab OR 'adolescence':kw,ti,ab OR 'adolescents, female':kw,ti,ab OR 'adolescent, female':kw,ti,ab OR 'female adolescent':kw,ti,ab OR 'female adolescents':kw,ti,ab OR 'adolescents, male':kw,ti,ab OR 'adolescent, male':kw,ti,ab OR 'male adolescent':kw,ti,ab OR 'male adolescents':kw,ti,ab OR 'youth':kw,ti,ab OR 'youths':kw,ti,ab OR 'teens':kw,ti,ab OR 'teen':kw,ti,ab OR 'teenagers':kw,ti,ab OR 'teenager':kw,ti,ab |  |
| #5 | 'randomized controlled trial':ab,ti OR 'controlled clinical trial':ab,ti OR randomized:ab,ti OR placebo:ab,ti OR 'clinical trials as topic':ab,ti OR randomly:ab,ti OR trial:ab,ti OR prospective:ab,ti OR retrospective:ab,ti | 5,651,524 |
| #6 | #1 AND #2 AND #3 AND #4 AND #5 | 1,051 |

| Web Of Science | | |
| --- | --- | --- |
| No. | Query | Results |
| #1 | TS=(ethanol lock OR citrate lock OR hydrochloric acid lock OR taurolidine lock OR taurolock OR urokinase OR lock solution OR flush solution OR flush OR flushing OR antibiotic OR antibiotics OR lock OR locks OR locking OR lock therapy OR lock treatment OR lock regimen OR lock technique OR antibiotic lock OR antibiotic locks OR antibiotic-lock OR antibiotic-locks OR antibiotic locking OR antibiotic-locking OR antibiotic lock treatment OR ALT OR Antimicrobial lock technique OR catheter lock) | 2,124,601 |
| #2 | TS=(central venous catheters OR Catheter, Central Venous OR Catheters, Central Venous OR Venous Catheter, Central OR Venous Catheters, Central OR Central Venous Catheter OR Central vascular access device OR CVCs OR catheter-related infections OR catheter related complications OR broviac OR port-a-cath OR port a cath OR implantable port OR implantable ports OR implantable catheter OR implantable catheters OR totally implantable access port OR totally implantable access ports OR Implantable Catheters OR Implantable Catheter OR Vascular Access Ports OR Vascular Access Port OR TIAP OR TIAPs OR hickman OR catheterization central venous OR tunnelled central venous catheter OR TCVC OR catheterization central venous OR peripherally inserted central Catheter OR picc OR central venous line OR central venous device OR central venous access device OR CVAD OR CVC OR Indwelling Catheter OR Indwelling Catheters OR In Dwelling Catheters OR In Dwelling Catheters OR In-Dwelling Catheter OR Venous Reservoirs OR Venous Reservoir) | 151,080 |
| #3 | TS=(Catheter-Related Infections OR Catheter-Related Infection OR Catheter Related Infections OR Infection, Catheter-Related OR Infections, Catheter-Related OR Catheter-Associated Infections OR Catheter-Associated Infection OR Catheter Associated Infections OR Infection, Catheter-Associated OR Infections, Catheter-Associated OR Catheter Related Infection OR Infection, Catheter Related OR Infections, Catheter Related OR Related Infection, Catheter OR Related Infections, Catheter OR Catheter Associated Infection OR Associated Infection, Catheter OR Associated Infections, Catheter OR Infection, Catheter Associated OR Infections, Catheter Associated OR catheter-related bloodstream infection OR catheter-related bloodstream infections OR CRBSI OR CRBSIs OR catheter-related bacteremia OR catheter-related bacteremias OR catheter-related bacteraemia OR catheter-related bacteraemias OR BSI OR CABSI OR CABSIs OR catheter associated bloodstream infection OR catheter-associated bloodstream infections OR catheter related complications OR Sepsis OR bacteremia OR bacteremias OR bacteraemia OR infection OR infections OR line infection OR line infections OR bloodstream infection OR bloodstream infections OR infectious diseases OR Pyemia OR Pyemias OR Pyohemia OR Pyohemias OR Pyaemia OR Pyaemias OR Septicemia OR Septicemias OR Septicaemia OR Septicaemias OR Severe Sepsis OR CVAD-associated grampositive infections OR central line–associated blood stream infection OR Contamination) | 7,530,291 |
| #4 | TS=(Infant OR Infan OR Infants OR premature infants OR Infants, Newborn OR Newborn Infant OR Newborn Infants OR Neonate OR Neonates OR Newborns OR Newborn OR babies OR baby OR Neonat OR Perinat OR postnat OR Schoolchild OR school child OR Child OR Children OR Preschool Child OR Children, Preschool OR Preschool Children OR Kid OR kids OR pediatrics OR pediatric OR Pediatric patients OR adoles OR Adolescent OR Adolescents OR Adolescence OR Adolescents, Female OR Adolescent, Female OR Female Adolescent OR Female Adolescents OR Adolescents, Male OR Adolescent, Male OR Male Adolescent OR Male Adolescents OR Youth OR Youths OR Teens OR Teen OR Teenagers OR Teenager) | 9,270,659 |
| #5 | TS= (randomized controlled trial OR controlled clinical trial OR Randomized OR placebo OR clinical trials as topic OR randomly OR Trial OR Prospective OR Retrospective) | 8,325,000 |
| #6 | #1 AND #2 AND #3 AND #4 AND #5 | 1,805 |
