# Supplementary material for: The efficacy of taurolidine lock solution in preventing catheter-related bloodstream infections in pediatrics: a systematic review and meta-analysis
Source: Front Pharmacol. 2026 Oct 21;17:1938862. doi: 10.3389/fphar.2026.1938862 (PMC13732722; doi:10.3389/fphar.2026.1938862)
Supplement: Supplementary file 2 [file Table2.docx]

**Supplementary Material 2.** Studies Excluded After Full-Text Assessment

| **Author/ year** | **Title** | **Exclusion reasons** |
| --- | --- | --- |
| Handrup, M. M et al. 2011 | Biofilm formation in long-term central venous catheters in children with cancer: a randomized controlled open-labelled trial of taurolidine versus heparin | Companion report involving participants from the trial reported by Handrup et al. (2013), with a primary focus on catheter biofilm formation. Not included as an independent dataset to avoid double-counting participants and overlapping outcome data. |
| Handrup, M. M et al. 2012 | Central Venous Catheters and Catheter Locks in Children With Cancer: A Prospective Randomized Trial of Taurolidine Versus Heparin | Report of the same trial included as Handrup et al. (2013). The trial was represented once in the meta-analysis using the retained primary publication to avoid duplicate inclusion. |
| Lambe C et al. 2016 | The impact of taurolidine-citrate locks implementation in a large pediatric cohort on home parenteral nutrition | Overlapping patient cohort with the included Lambe et al. (2018) study. The 2018 publication was retained as the data source for the relevant comparison, and the overlapping data from the 2016 report were not included separately. |
| Akyüz, C et al. 2010 | prophylactic taurolidine use in central venous catheters of pediatric cancer patients: a prospective randomized study from single center | Only abstract-level information was available; the full study report could not be obtained. The available information was insufficient to verify the methodological details and extract the data required for quantitative synthesis. |
| I. Savarese et al. 2024 | Use of 2% taurolidine lock solution for treatment and prevention of catheter-related bloodstream infections in neonates: a feasibility study | Insufficient extractable comparative data to calculate an incidence rate ratio and its standard error. Corresponding infection-event counts and catheter-days for the eligible intervention and comparator groups, or a directly usable IRR with its confidence interval, were not available. |
| Demirok, A et al. 2024 | Catheter salvage or removal in catheter‐related bloodstream infections with Staphylococcus aureus in children with chronic intestinal failure receiving home parenteral nutrition and the use of prophylactic taurolidine catheter lock solution: A descriptive cohort study | Descriptive cohort evaluating the management of Staphylococcus aureus CRBSI in patients routinely receiving prophylactic taurolidine locks. No eligible comparison between taurolidine and a non-taurolidine locking strategy was provided; therefore, a comparative IRR could not be estimated. |
