# Supplementary material for: The efficacy of taurolidine lock solution in preventing catheter-related bloodstream infections in pediatrics: a systematic review and meta-analysis
Source: Front Pharmacol. 2026 Oct 21;17:1938862. doi: 10.3389/fphar.2026.1938862 (PMC13732722; doi:10.3389/fphar.2026.1938862)
Supplement: Supplementary file 3 [file Table3.docx]

**Supplementary Material 3.** Characteristics of Catheter Lock Protocols

| Study / year | Exact formulation and concentration | Lock volume | Dwell time | Administration / lock frequency | Catheter type | Infection definition | Taurolidine events | Taurolidine catheter-days | Control events | Control catheter-days | Reported IRR | Reported 95% CI |
| --- | --- | --- | --- | --- | --- | --- | --- | --- | --- | --- | --- | --- |
| Simon 2008 | **Taurolidine: Taurolidine 1.35% + sodium citrate 4%.**  **Control: Heparin 200 IU in 2 mL of 0.9% saline.** | **2 mL per lumen** | **NA** | **Double-lumen Broviac/Hickman devices were re-locked 1–2 times weekly.**  **Ports were not routinely flushed or re-locked while not in use.** | **Totally implanted ports or double-lumen Broviac/Hickman catheters.** | **CRBSI** | **3** | **6,705** | **14** | **6,086** | **NA** | **NA** |
| Dümichen 2012 | **Taurolidine: Taurolidine 1.35% + sodium citrate 4%.**  **Control: Heparin 100 IU/mL in saline.** | **Catheter-specific filling volume** | **NA** | **Instilled after each treatment cycle when the catheter was not continuously used.**  **No lock was applied during continuous catheter use.** | **Tunneled single, double or triple lumen Broviac/Hickman catheters.** | **CRBSI** | **2** | **6,576** | **9** | **7,233** | **NA** | **NA** |
| Handrup 2013 | **Taurolidine:** **Taurolidine 1.35% + sodium citrate 4% + heparin 100 IU/ mL**  **Control: Heparin 100 IU/mL (250 IU in 2.5 mL).** | **2.5 mL per lumen.** | **NA** | **Instilled after catheter use / after each treatment cycle.** | **TE or TID** | **CRBSI** | **7** | **NA** | **26** | **NA** | **0.260** | **0.090-0.610** |
| Ince 2014 | **Taurolidine: taurolidine 1.35% + sodium citrate 4%.**  **Control: Diluted heparin 100 IU/mL** | **NA** | **NA** | **NA** | **Mono-lumen TIAPs; Deltec or Polysite devices.** | **CRBSI** | **18** | **NA** | **16** | **NA** | **NA** | **NA** |
| Hulshof 2017 | **Taurolidine: Taurolidine 2%.**  **Control: Heparin lock.** | **NA** | **NA** | **Taurolidine could be used when parenteral nutrition infusion was <23.5 h/day,** | **NA** | **CRBSI** | **8** | **2,598** | **41** | **2,747** | **NA** | **NA** |
| Lambe 2018 | **Taurolidine: Taurolidine 1.35% + sodium citrate 4%.**  **Control: normal saline** | **NA** | **NA** | **between 2 cycles of parenteral infusions varying from 8 to 60 hours (median 12 hours)** | **NA** | **CRBSI** | **5** | **20,403** | **89** | **99,774** | **NA** | **NA** |
| Clark 2019 | **Taurolidine: Taurolidine 1.34% + sodium citrate 4%.**  **Control: Heparin 10 IU/mL when inactivity was <3 days or 100 IU/mL when inactivity was >3 days.** | **2 mL for a tunneled cuffed CVAD and 4 mL for a totally implanted device.** | **At least 6 hours and up to 28 days.** | **Daily.** | **tunneled cuffed CVADs or totally implanted devices.** | **CLABSI** | **5** | **10,359** | **39** | **7,077** | **NA** | **NA** |
| Chong 2020 | **Taurolidine: Taurolidine 1.35% + sodium citrate 4%**  **Control: Heparin lock.** | **NA** | **At least 8 hours.** | **NA** | **Hickman catheters, implantable ports or PICCs.** | **CLABSI** | **NA** | **NA** | **NA** | **NA** | **0.200** | **0.110-0.320** |
| van den Bosch 2024 | **Taurolidine: Taurolidine 1.35% + sodium citrate 4% + heparin 100 IU/mL**  **Control: Heparin 100 IU/mL** | **0.8–1.5 mL per lumen, by device:** **Babyport 1.0 mL; low-profile/standard TIVAP 1.5 mL; single-lumen external tunneled catheter 1.0 mL; double-lumen 1.0/1.0 mL; triple-lumen 1.0/0.8/0.8 mL** | **Remained in place until the next catheter use.** | **After each treatment cycle, no more than once weekly.** | **external tunneled catheters or totally implanted venous access ports.** | **CLABSI** | **21** | **18,398** | **26** | **18,559** | **NA** | **NA** |

**CRBSI**, catheter-related bloodstream infection; **CLABSI**, central line-associated bloodstream infection; **CAVD**, Central venous access device; **NA**, not available; **PICCs**, peripherally inserted central catheters; **TIAPs**, totally implantable access ports; **TE**, tunneled CVC with external lines; **TID**, total implantable device.
